# Is integrated private-clinic based early child development care effective? A clustered randomised trial in Pakistan

**DOI:** 10.3399/bjgpopen18X101593

**Published:** 2018-06-27

**Authors:** Muhammad Amir Khan, Syeda Somyyah Owais, Shazia Maqbool, Sehrish Ishaq, Haroon Jehangir Khan, Fareed A Minhas, Joseph Hicks, Muhammad Ahmar Khan, John D Walley

**Affiliations:** 1 Chief Coordinating Professional, Association for Social Development, Islamabad, Pakistan; 2 Project Manager, Association for Social Development, Islamabad, Pakistan; 3 Professor, Development & Behavioural Pediatrics Department, Institute of Child Health and The Children Hospital, Lahore, Pakistan; 4 Project Coordinator, Association for Social Development, Islamabad, Pakistan; 5 Director, NCD & Mental Health, Directorate General of Health Services, Punjab, Pakistan; 6 Head, The Institute of Psychiatry, Rawalpindi, Pakistan; 7 Senior Medical Statistician, Health Nuffield Centre for International Health and Development, University of Leeds, Leeds, UK; 8 Research Coordinator, Association for Social Development, Islamabad, Pakistan; 9 Clinical Professor of International Public, Nuffield Centre for International Health and Development, University of Leeds, Leeds, UK

**Keywords:** primary care, primary health care, general practice, Pakistan, child development, nutrition, depression

## Abstract

**Background:**

In Pakistan, high prevalence of delays in early child development (ECD) is associated with poverty and lack of mothers’ caregiving skills. GP clinics, the main sources of care in poor urban localities, lack quality ECD care delivery. A contextualised intervention was developed and tested to enable GPs to deliver clinic-based, tool-assisted ECD counselling of mothers on a quarterly basis.

**Aim:**

To assess the effectiveness of delivering a contextualised ECD mother-counselling intervention.

**Design & setting:**

Clustered randomised controlled trial, in poor urban localities of Pakistan. Locality clusters were allocated to intervention and control arm using simple randomisation.

**Method:**

A total of 2327 mother–child pairs were recruited at 32 GP clinics, one from each cluster-locality; 16 GP clinics per arm. The clinic-based counselling intervention covering child stimulation, nutrition, and maternal mental health was delivered mainly by clinic assistants to mothers at ≤6 weeks, and 3, 6, and 9 months of child age. At 12 months of child age, each mother–child pair was assessed for the primary outcome, that is, delays in the five development domains (determined by Ages and Stages Questionnaire-3 [ASQ-3] score); and secondary outcomes, namely the prevalence of stunting and maternal depression (determined by Patient Health Questionnaire-9 [PHQ-9] score). The outcome assessors were blinded to the cluster–arm allocation. Outcome analyses were calculated on cluster-level.

**Results:**

At 12 months, the number of children with delay in two or more development domains was significantly lower in the intervention arm (-0.17 [95% confidence interval {CI} = -0.26 to -0.09]; *P*<0.001) compared to the control arm. The difference in the prevalence of child stunting and maternal depression were also significant at -0.21% (95% CI = -0.30 to -0.13; *P*<0.001) and -0.23% (95% CI = -0.29 to -0.18; *P* = 0.000) respectively.

**Conclusion:**

Contextualised ECD care, when delivered at GP clinics in poor urban localities, can effectively reduce the developmental delays during the first 12 months of the child's life.

## How this fits in

In Pakistan, the GP clinics are main source of primary health care for people living in poor urban localities. These clinics currently lack quality ECD care to enable mothers of young children to achieve better physical and mental development during the first year of the child's life. This study aimed to develop and test a set of simplified implementation guidelines and tools that can be used effectively, by modestly educated (but trained) staff, to deliver integrated ECD care at GP clinics.

## Introduction

The first 1000 days of life impacts a child’s survival, growth, and development; high rate of growth during this time leads to rapid responsiveness to the surrounding environment.^1^ Neural pathways for communication, problem-solving, motor movement, and emotional understanding are developed during this period.^2–4^ Crucial mediators of infant outcomes include the quality of parents’ responsive feeding and caregiving skills, and maternal health to enable the delivery of those skills. Approximately 8 million children in Pakistan fail to meet their developmental potential within their first 5 years, signifying that 20–40% children under 5 years of age are disadvantaged.^5^ With a stunting prevalence of 43.7%,^6^ these disadvantaged children are unable to gain subsequent skills or education, consequently fueling the cycle of poverty.^7^

ECD is often helped by age-appropriate learning opportunities and activities, created by caregivers to encourage development of language and problem-solving skills. The early social-emotional development of a child is also dependent on their caregiver’s positive emotionality, responsiveness, and sensitivity during caregiver–child interactions.^8,9^ Lack of these caregiver–child interactions and learning opportunities can harm healthy growth and brain development potential.^10^ In lower and middle-income countries, poor parental stimulation has been shown to be associated with decreased child development.^11^

Mothers are often the primary caregivers, and often need help and support to develop basic skills for providing a responsive and stimulating environment for their child.^12^ Psychosocial factors and lack of counselling can influence a mother’s ability to create this optimal environment.^13^ Developmental delay in children is often made worse by mothers having low levels of literacy, knowledge, support, and guidance.^14^ Moreover, these developmental problems are exacerbated with more severe and chronic levels of maternal depression,^15–17^ which are high in Pakistan (24–36%).^18^

Interventions promoting mother–child interactions through counselling, delivered at health centres, have been shown to benefit children’s brain development.^19,20^ In Pakistan, people in poor urban areas usually seek primary care at private clinics due to the lack of public primary care services and the difficulty in accessing tertiary care hospitals due to distance, affordability, and high demand. Private clinics are often unregulated and vary in the services provided, and the existing maternal and child health services do not cater to early child brain development. Therefore, a district-steered intervention was developed, consisting of an ECD package integrated at private GP clinics in poor urban communities. Objectives included evaluating the effectiveness of the intervention at:

- reducing child developmental delays in two or more domains by 20% (from 20% to 16%);
- decreasing the prevalence of stunting (below 2 standard deviation height for age Z-score) from 39% to 35%; and
- decreasing the prevalence of maternal depression 36% to 29%.

## Method

The methods of the trial have already been published in detail^21^ but are briefly described below.

### Study design and randomisation

A pragmatic, parallel arm, cluster randomised controlled trial was conducted to measure the effectiveness of an integrated ECD package at private clinics in poor urban localities (one clinic per locality cluster) in Pakistan. Randomising individual participants, including keeping the participants blinded to treatment allocation, was considered too complex for clinic staff to manage and therefore cluster randomisation was chosen. A total of 32 clusters were randomised to the intervention and control arms in a 1:1 ratio. Simple randomisation of selected clusters was performed by drawing clusters from a hat. This was done jointly by members of the research organisation and the provincial Directorate General of Health Services (DGHS) health directorate. Randomisation of the clinic-clusters in to intervention and control arm was followed by consented recruitment of mother–child pairs for the trial.

### Setting

The metropolitan districts of Lahore and Rawalpindi were selected as study sites, in consultation with the provincial DGHS. In Pakistan, each district is divided into smaller population units called union councils, with an average population of 15 000–25 000. There are 151 and 170 union councils in Lahore and Rawalpindi, respectively. Like most Pakistani urban settings, Lahore and Rawalpindi have a network of qualified GPs’ clinics. A private GP clinic usually includes a qualified doctor and a clinic assistant. The clinic assistant is usually a local male, with 10–12 years of schooling but no formal paramedic training.^22^ With on-the-job training provided by the clinic doctor, the assistant’s usual responsibilities are dispensing medicine, offering patient education, and performing other non-clinical tasks as necessary.

The two district health offices were asked to enlist 32 relatively poor union councils (16 in each district), based on available socioeconomic data (such as roads, housing, water supply, sanitation, and per capita income), to determine the lowest socioeconomic localities. Private clinics in the selected union councils were mapped, and two clinics in each union council were shortlisted based on relative maximum number of years' establishment, higher patient load, and availability of maternal and child health care. Then one clinic was selected in each union council based on its willingness to participate, and the district health office's perception of the likelihood of the clinic staff's cooperation. A formal written consent (in the form of memorandum of understanding) was obtained from each selected clinic’s doctor, through the respective district health office, before randomisation.

### Sample size

At least 2112 mother–child pairs were required (1056 in each arm), with an average of 66 mother–child pairs in each of the 32 clusters (assuming equal cluster sizes), to detect an absolute difference of ≥20% in development delay, assuming a baseline suspected development delay of 33%,^23^ with 80% power, a 5% level of significance, and assuming a 10% loss to follow-up.^24^ Considering assumed mother–child caseload at a clinic, inclusion of 32 clinics was recommended to get the required number of mother–child pairs recruited within the stipulated time.

### Recruitment of mother–child pairs

Each (self or peer-referred) mother visiting the clinic with a child aged <40 days was assessed by the clinic assistant for inclusion eligibility based on the child being ≥2.5 kg at birth, free of congenital abnormalities, and without a history of delayed cry at birth and/or seizures.

The clinic assistant informed each eligible mother about the trial and asked for her consent to participate. Those who consented were registered in the trial and offered ECD care as per the respective trial arm protocol. In case of refusal, the reason for refusal was documented (where possible) and data were not used for trial purposes. The mother–child pair retained the right to withdraw participation in the trial at any given point. The mother–child registration was stopped when the required number of registrations in the respective trial arm was achieved.

### Early childhood development intervention

The core intervention, delivered mainly by clinic assistants at private clinics, focused on quarterly counselling of mothers via a specifically designed flip-book. The tool-assisted counselling supplemented the mother’s ability to promote age-appropriate activities for ECD and improved child nutrition, and to manage her own depression. The content of the quarterly mother counselling sessions was agreed through technical expert deliberations, guided by international guidelines^3,25^ and informed by in-country experiences. The contents were then transformed into a set of contextualised pictures and messages packaged as a flip-book. The contents of each counselling session were designed to take ≤10 minutes. Tailor-made training programmes for doctors and clinic assistants were also developed and used to enable the clinic staff to deliver the ECD care and maintain records.

The registered mother–child pairs were required to visit the clinic every 3 months to get assessed and counselled by the clinic assistant, when the child was aged 3, 6, and 9 months. In case of suspected child development delay or maternal depression, the doctor assessed the mother and/or child and advised further management, including referral for specialist care (see Box 1 for more details of the intervention).

**Box 1.** Summary of early child development care package (adapted)^21^

**Intervention clinics**	Care tasks delivered by clinic assistant: Ten minute tool-assisted, structured counselling session of mother (using flip-book), covering early childhood nutrition, early childhood development, and maternal mental health Provision of take-home brochure to mothers to revise key counselling messages for each child development milestone Follow-up of mother–child pairs in clinic at 3, 6, and 9 months of child age (including text message or telephone reminder, if required). Monitoring and screening of child growth, and child and maternal mental health Referral to clinic doctor in cases of malnourishment, signs of child development delay, or maternal depression
	Care task delivered by clinic doctor: Assessment and treatment (including referral to specialist) of childhood nutrition, development, or maternal depression
	Clinic assistants trained on: Conduct of structured counselling session using the flip-book Administration of the first two questions of the Patient Health Questionnaire-9 (known as PHQ-2) Measurement and recording of child length and weight
	Private GPs trained on: Clinical management of children with malnutrition and developmental delay in the private clinic setting and specialist referral Diagnosis of maternal depression PHQ-9
**Intervention and control clinics**	District health endorsement of early child development care at clinics Training on measurement of child weight and height, and record-keeping Recruitment of community advocates for referral and registration of mother–child pairs at clinics
**Control clinics**	Provision of usual routine care

### Control arm receiving routine care

The control clinics were also enabled to recruit mother–child pairs as per the trial protocol by applying the same inclusion criteria. This was done to minimise differences at registration. The control clinics were advised to continue with their usual routine mother and child care practices. The current mother–child care at private clinic is curative-oriented (that is, responding to an ailment) rather than health promotion-oriented. In ECD care, the private clinics do not provide any child development counselling, instead responding to any complaint that is reported or noted.

Control clinics were not provided with the tool (flip-book) or the training on skills to counsel mothers for ECD, child nutrition, and maternal depression. As in the intervention arm, the control clinics were provided with digital weighing scales and infantometers, to ensure quality and comparability of child anthropometric measurements.

### Outcomes

The primary outcome was a binary indicator of whether or not a child had reduced delay in two or more child development domains. The five development domains measured at 12 months of child age were: communication, gross motor, fine motor, problem-solving, and personal-social. A translated version (in Urdu) of ASQ-3 was used by a pair of trained assessors to interview mothers at clinics. The assessors were blinded to the mother–child allocation. The mother interview was supplemented by an on-site observation (using a specially designed checklist) to measure the child’s achievement in the five development domains at each clinic during outcome measurements. The addition of on-site observation was mainly to compare the lay (mother's) and technical (assessor's) assessments, and to explore its added value for similar clinic-based measurements in the future. To minimise age-variation, measurements were taken from children when they were aged 11 months–﻿12 months and 29 days. The child's development (and other) outcomes were measured at an individual level, whereas the analysis for primary and secondary outcomes were done at the cluster level.

The secondary outcomes measures were: occurrence of underweight and stunting in children aged 12 months, and depression in mothers of children aged 12 months, as measured with the PHQ-9 (detailed results on a set of secondary outcome measures will be separately published elsewhere).

### Statistical analysis

Data for each mother–child pair were analysed using SPSS (version 21.0). All clusters were analysed according to their original allocations (that is, intention to treat basis), and only complete cases were analysed where baseline and outcome measurements were found not missing (including covariates for adjusted analyses). All outcomes were collected at the individual level but analysed at the cluster-level, using methods suitable for cluster trials.^26^ Inference was based on formal hypothesis testing at the 5% alpha level. For the crude analyses, cluster-level summary measures were calculated from the individual-level outcomes, either as proportions (for the primary outcome and all binary secondary outcomes) or as means (for all continuous secondary outcomes). Then an estimate of the intervention effect was calculated either as a risk difference or a mean difference (between the intervention and control arms) for binary or continuous outcomes respectively. An independent *t*-test was then used to estimate 95% confidence intervals and *P*-values for these treatment effect estimates.

For the adjusted analyses, a two-stage process was used.^26^ First, for the primary outcome and all binary secondary outcomes, a logistic regression was initially fitted to the individual-level outcome data, controlling for covariates of interest except for the treatment effect. All outcomes were adjusted for the following covariates at registration: child age at registration (in days), child age at outcome measurement (in days), sex, weight for age, stunting, family structure, number of siblings, number of siblings aged <5 years, and maternal age and education. Covariate-adjusted, cluster-specific difference residuals were then calculated based on the model-predicted values and observed outcomes. Secondly, covariate-adjusted risk differences and their associated 95% confidence intervals and *P*-values were calculated based on the difference residuals, using a *t*-test as above. For the continuous secondary outcomes, a similar two-stage process was used, except that a linear regression was used to calculate the difference residuals, and covariate-adjusted treatment effects were estimated as mean differences.

## Results

**Figure 1.**
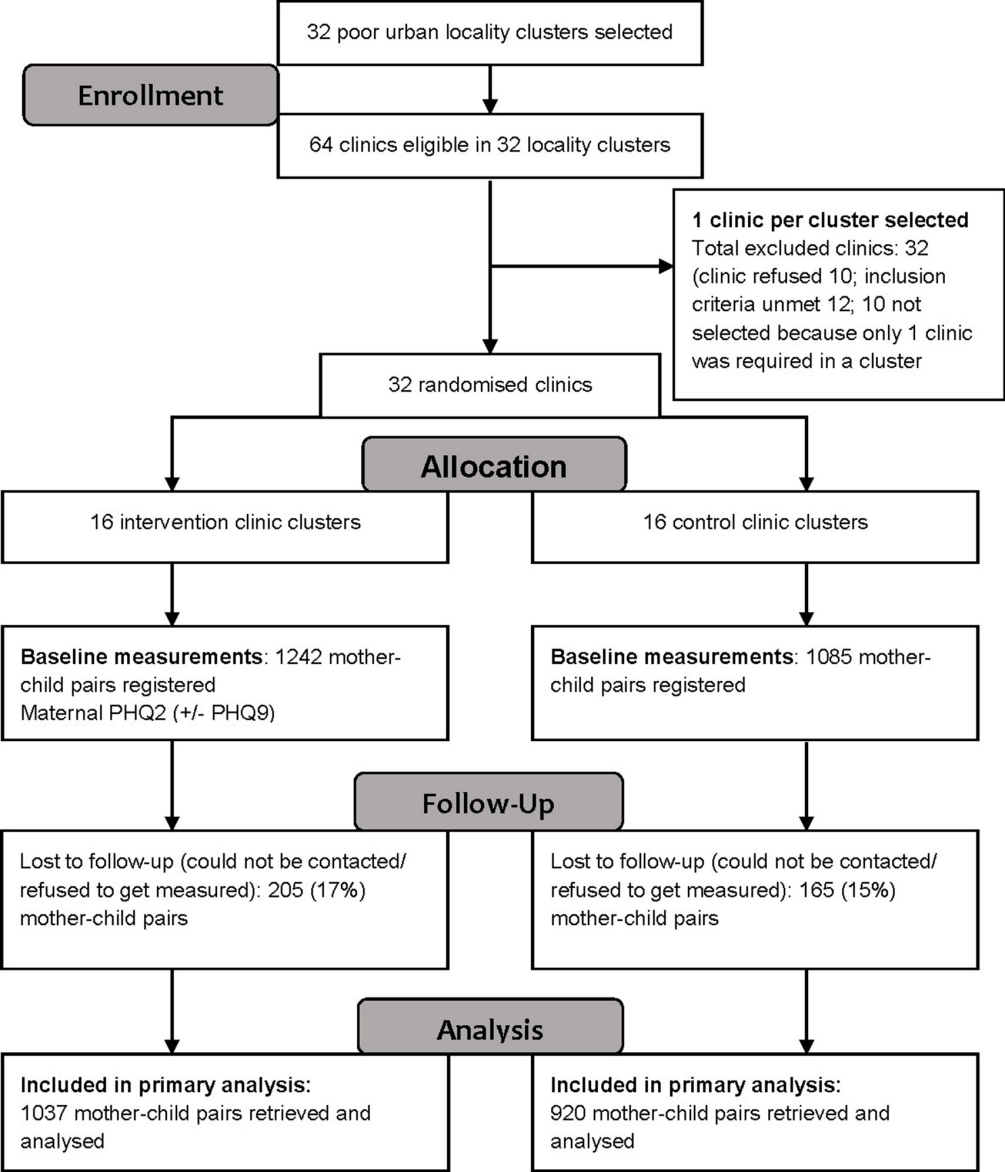
Trial flow diagram.

Between April 1 2014–September 30 2016, the trial recruited 2327 mother–child pairs (1242 in the intervention arm and 1085 in the control arm) for in-depth assessment for ECD (Figure 1). Out of the 2327 enrolled mother–child pairs, 1957 (84%; intervention, *n* = 1037/1242; control; *n* = 920/1085) were successfully followed-up for outcome measurements when the child reached 12 months of age. There were no substantial differences in any of the baseline variables between the intervention and control arms (**Table 1**).

**Table 1.** Baseline characteristics of clusters (total, *n* = 32; intervention, *n =* 16; control, *n* = 16) and participants (total, *n* = 2327; intervention, *n* = 1242; control, *n* = 1085)

Characteristics	Intervention	Control
**Clusters, *n***	16	16
Average cluster size	77.6 (±50.7)	67.8 (±59.8)
**Participants, *n* (%)**	1242 (53.4)	1085 (46.6)
**Maternal characteristics**		
Mean age, years (SD)	27.1 (±3.45)	27.1 (±3.15)
Mean education, years (SD)	8.3 (±3.9)	8.6 (±3.8)
Mean number of children^a^ (SD)	2.65 (±1.37)	2.67 (±1.24)
Mean number of children aged <5 years^a^ (SD)	1.65 (±0.60)	1.65 (±0.61)
**Family structure**		
Joint, *n* (%)	824 (79.5)	694 (75.4)
Nuclear, *n* (%)	213 (20.5)	226 (24.6)
**Child characteristics**		
**Sex**		
Male	671 (54)	582 (53.6)
Female	571 (46)	503 (46.4)
**Mean age, days (SD)**		
Male	15.3 (±13.8)	10.6 (±14.2)
Female	15.9 (±14.5)	9.7 (±14.0)
**Mean height, cm**		
Male	50.91 (±3.91)	50.09 (±3.33)
Female	50.07 (±3.68.)	49.44 (±3.01)
**Mean weight, kg**		
Male	3.28 (±0.52)	3.25 (±0.68)
Female	3.20 (±0.50)	3.07 (±0.47)
**Stunting^b^, *n* (%)**		
Total	310 (25.0)	271 (25.0)
Male	165 (24.6)	145 (24.9)
Female	145 (25.4)	126 (25.0)

^a^Excluding the child registered in the trial.

^b^Defined as moderate and severe (below minus two SDs from median height for age of reference population).

CM = centimetres. KG = kilograms. SD = standard deviation.

The adjusted risk differences of children with delay in two or more domains (the primary outcome) was significantly lower in the intervention arm as compared to the control arm (-0.17 [95% CI = -0.26 to -0.09; *P*<0.001]) as shown in **Table 2**. The intervention arm also had a significantly lower proportion of children with delay in each of the five development domains: communication (-0.09 [95% CI = -0.17 to -0.02; *P* = 0.018]), gross motor (-0.13 [95% CI = -0.19 to -0.08; *P*<0.001]), fine motor (-0.12 [95% CI = -0.21 to -0.04; *P* = 0.009]), problem-solving (-0.05 [95% CI = -0.90 to -0.02; *P* = 0.004]), and personal-social (-0.07 [95% CI = -0.14 to -0.02; *P* = 0.043]).

**Table 2.** Primary outcome risk differences of delays in two or more domains and delays in early development domains, using mother ASQ-3, child age 12 months (total, *n* = 1957; intervention, *n* = 1037; control, *n* = 920). All outcomes analysed at cluster level.

	Intervention (clusters, *n* = 16)	Control (clusters, *n* = 16)	Crude intervention-control difference (95% CI); *P*-value^a^	Adjusted intervention-control difference (95% CI); *P*-value^a^
	Proportion outcome (95% CI)			
**ECD outcome**				
Two or more delays^b^	0.14 (0.11 to 0.17)	0.32 (0.24 to 0.41)	-0.18 (-0.27 to -0.10); <0.001	-0.17 (-0.26 to -0.09); 0.001
Communication	0.18 (0.14 to 0.22)	0.28 (0.21 to 0.35)	-0.10 (-0.18 to -0.03); 0.011	-0.09 (-0.17 to -0.02); 0.018
Gross motor	0.08 (0.06 to 0.11)	0.22 (0.17 to 0.27)	-0.14 (-0.19 to -0.08); <0.001	-0.13 (-0.19 to -0.08); <0.001
Fine motor	0.11 (0.08 to 0.14)	0.24 (0.15 to 0.32)	-0.13 (-0.22 to -0.04); 0.007	-0.12 (-0.21 to -0.04); 0.009
Problem-solving	0.03 (0.02 to 0.05)	0.09 (0.06 to 0.13)	-0.06 (-0.10 to -0.02); 0.005	-0.05 (-0.09 to -0.02); 0.004
Personal-social	0.22 (0.19 to 0.25)	0.29 (0.22 to 0.36)	-0.07 (-0.15 to -0.00); 0.048	-0.07 (-0.14 to -0.02); 0.043

^a^Data are proportions (95% CI). The analysis is adjusted for clustering and controlled for several covariates (sex of the child, child weight at registration, child height at registration, child age at registration and endline, maternal age, maternal education, number of additional children the mother has, number of additional children aged <5 years, and family structure) by logistic regression analysis.

^b^Overall ICC = 0.14, intervention arm ICC = 0.03 and control arm ICC = 0.12.^25^

ASQ-3 = Ages and Stages Questionnaire-3. CI = confidence interval. ECD = early child development. ICC = intracluster correlation.

The difference in the adjusted proportion of children with two or more delays, and delays in the five development domains, remained significant even when only on-site observation scores were used for the comparison (**Table 3**).

**Table 3.** Risk differences of delays in two or more domains and delays in early development domains, using on-site observations (total, *n* = 1957; intervention, *n* = 1037; control, *n* = 920). All outcomes are analysed at cluster-level.

	Intervention (clusters, *n* = 16)	Control (clusters, *n* = 16)	Crude intervention-control difference (95% CI); *P*-value	Adjusted intervention-control difference (95% CI); *P*-value
	Proportion outcome (95% CI)			
**Observation outcome**				
Two or more delays	0.15 (0.12 to 0.18)	0.35 (0.26 to 0.45)	-0.20 (-0.30 to -0.10); 0.001	-0.20 (-0.30 to -0.10); 0.001
Communication	0.20 (0.15 to 0.23)	0.29 (0.21 to 0.37)	-0.10 (-0.18 to -0.01); 0.032	-0.09 (-0.18 to 0.00); 0.042
Gross motor	0.12 (0.05 to 0.17)	0.25 (0.16 to 0.33)	-0.13 (-0.23 to -0.02); 0.015	-0.13 (-0.23 to -0.02); 0.015
Fine motor	0.09 (0.06 to 0.12)	0.24 (0.13 to 0.34)	-0.14 (-0.25 to -0.04); 0.010	- 0.14 (-0.24 to -0.04); 0.009
Problem-solving	0.06 (0.05 to 0.07)	0.13 (0.09 to 0.16)	-0.06 (-0.10 to -0.02); 0.003	-0.01 (-0.07 to -0.05); 0.003
Personal-social	0.22 (0.17 to 0.26)	0.38 (0.28 to 0.48)	-0.16 (-0.27 to -0.05); 0.005	−0.16 (-0.26 to -0.05); 0.004

The analysis is adjusted for clustering and controlled for several covariates (sex of the child, child weight at registration, child height at registration, child age at registration and endline, maternal age, maternal education, number of additional children the mother has, number of additional children aged <5 years, and family structure) by logistic regression analysis.

CI = confidence interval.

Average achievement in each of five development domains (mean score) was also found to be higher after adjusting for covariates in the intervention arm as compared to the control arm (**Table 4**).

**Table 4.** Mean differences in scores of early child development domains using mother's ASQ-3 responses, at child age 12 months (total, *n* = 1957; intervention, *n* = 1037; control, *n* = 920). All outcomes are analysed on cluster-level.

	Intervention (clusters, *n* = 16)	Control (clusters, *n* = 16)	Crude intervention-control difference (95% CI); *P*-value*^b^*	Adjusted intervention-control difference (95% CI); *P*-value*^b^*
	Mean outcome (95% CI)			
Communication	43.05 (41.18 to 44.91)	33.61 (31.34 to 35.88)	9.43 (6.62 to 12.25); <0.001	7.44 (3.95 to 9.73); <0.001
Gross motor	43.94 (41.69 to 46.20)	36.47 (34.24 to 38.70)	7.47 (4.43 to 10.50); <0.001	5.51 (2.71 to 8.29); <0.001
Fine motor	47.39 (45.07 to 49.70)	43.30 (40.95 to 45.65)	4.09 (0.93to 7.25); 0.013	2.84 (0.32 to 5.37); 0.029
Problem-solving	46.95 (44.54 to 49.35)	40.68 (39.11 to 42.24)	6.27 (3.50 to 9.03); <0.001	4.80 (2.65 to 6.95); <0.001
Personal-social	38.62 (36.67 to 40.57)	32.28 (30.26 to 34.30)	6.34 (3.65 to 9.03); <0.001	4.80 (2.65 to 6.95); <0.001

Data are mean of scores in each development domain (95% CI) on the ASQ-3 mother responses. The analysis is adjusted for clustering and controlled for several covariates (sex of the child, child weight at registration, child height at registration, child age at registration and endline, maternal age, maternal education, number of additional children the mother has, number of additional children aged <5 years, and family structure) by logistic regression analysis.

ASQ-3 = Ages and Stages Questionnaire-3. CI = confidence interval.

Measurement through mother-interviewing and on-site assessing (by a qualified assessor) showed around 90% agreement between the two sets of outcome measurements (Table 5). As shown in Table 5, on-site observation (by assessor) was not able to verify the mother-reported achievement in about 5% cases; whereas in 3% and 6% of intervention and control children respectively, the on-site observation was able to identify an achievement missed by the mother.

**Table 5.** Agreement between mother-response and on-site observation by assessor (total, *n* = 1957; intervention, *n* = 1037; control, *n* = 920)

	Agreement^a^		Disagreement type 1^b^		Disagreement type 2^c^	
**Development domains**	**Intervention, n (%)**	**Control, *n* (%)**	**Intervention, *n* (%)**	**Control, n (%)**	**Intervention, *n* (%)**	**Control, *n* (%)**
Communication	863 (83.2)	746 (81.1)	42 (4.1)	61 (6.6)	23 (2.2)	56 (6.1)
Gross motor	955 (92.1)	811 (88.2)	60 (5.8)	59 (6.4)	22 (2.1)	50 (5.4)
Fine motor	953 (91.9)	798 (86.7)	32 (3.1)	50 (5.4)	52 (5.0)	72 (7.8)
Problem-solving	990 (95.4)	821 (89.2)	36 (3.5)	63 (6.8)	11 (1.1)	36 (3.9)
Personal-social	879 (84.7)	738 (80.2)	89 (8.6)	104 (11.3)	69 (6.7)	78 (8.5)
Total measurements^d^	4640 (89.5)	3914 (85.0)	259 (5.0)	243 (5.3)	177 (3.4)	292 (6.3)

^a^Both mother-response scores (on ASQ-3) and assessor observation scores both conclude the child has delayed or achieved milestones for the specified domain (that is, reports tally).

^b^Type 1 disagreement: mother-response indicates milestone achievement, but observation scores suggest delay.

^c^Type 2 disagreement: mother-response indicates milestone delay, but observation scores suggest achievement.

^d^Out of total 5185 and 4600 observations in intervention and control, respectively (calculated using total number of measured children X5 [development domains])

ASQ-3 = Ages and Stages Questionnaire-3.

The percentage of children with stunting (below 2 standard deviation height for age Z-score), at 12 months of age, was significantly lower in intervention arm (13%) as compared to control arm (35%). The difference remained significant after adjusting for the baseline covariates (-0.21 [95% CI = -0.30 to -0.13; *P*=0.00]). The percentage of underweight children (below 2 standard deviation weight for age Z-score), at 12 months of age, was also lower (22%) in intervention arm as compared to control arm (27%); but the difference was not statistically significant. The percentage of mothers with depression (PHQ-9 score ≥5) was significantly lower in the intervention arm (5%) as compared to the control arm (30%), with an adjusted risk difference of (-0.23 [95% CI = -0.29 to -0.18]; *P* = 0.000).

## Discussion

### Summary

A clustered randomised controlled trial was conducted to investigate the effectiveness of delivering an integrated ECD care package at private clinics in poor urban settings in Pakistan. The results show that the intervention had significant benefits on all ECD domains, including a significant reduction in the proportion of delays in two or more development domains. The private clinic staff received minimal training, as well as material and monitoring support to deliver a contextualised ECD counselling and care package. This enabling for ECD required an investment of about US $80 per clinic, and each clinic can register an average of 100 mother–child pairs in a year. The 20% additional reduction in two or more child development delays (as found in the trial results) implies that an investment of US $4 at a clinic can potentially prevent a child from having two or more developmental delays at the age of 12 months.

### Strengths and limitations

The cluster randomisation method (picking numbers from a hat) was technically a less than optimal method for randomisation, but had an advantage of engaging the management staff of the respective health authority in the province. It was not possible to blind the careprovider and mothers to the counselling intervention, only the assessors. However, geographic separation of clusters, and registering mother–child pairs from within the catchment population of the respective clinic, were the measures taken to minimise the risk of possible cross-cluster contamination.

The loss to follow-up (16%) exceeded the expected 10%. Studies have indicated that an attrition of >20% poses serious threats to a study’s validity.^27,28^ It was not feasible to retrieve these lost mother–child pairs. However, comparison of recorded characteristics at baseline (that is, at registration) showed no significant difference between the mother–child pairs completing and defaulting the 12 months of ECD care.

The decision to measure only the combined (and not separated) effect of child stimulation, nutrition, and maternal depression on the child's development was made to keep the trial protocols and requirements feasible within the available resource constraints. The initial effectiveness and feasibility suggest considering a larger multi-arm trial for separately measuring the effect of each of the three intervention components under routine programme circumstances.

It is important to note that the intervention focusing on poor urban settlements, being served by private clinics, poses a challenge to the extrapolation of the results to the general population, especially to those living in rural parts of the country.

### Comparison with existing literature

The Pakistan Early Child Development Scale-up (PEDS) trial, in rural Sindh, developed and tested an intervention in which community-based 'lady health workers' were enabled to deliver ECD care during home visits of the each mother–child pair. The trial was meant to assess separate and combined effects of the mother offering child stimulation and nutrition care. Their child measurements at age 12 months, based on Bayley Scales, showed a significant combined effect of child stimulation and nutrition. The results of the present study's combined intervention are comparable with the PEDS trial results. Comparison of delays in five domains indicate that PEDS achievement was slightly lower in two domains, namely motor and communication (at 16% and 22% delays), and better in two domains, namely problem-solving and personal-social (at 2% and 19%, respectively).^29^

In Pakistan, there has been limited reported experience of using ASQ-3 to measure all five domains of child development.^30–32^ Similarly, clinic-based measurement of child development has not been tested before. In the present study, the caregiver responses were supplemented by on-site observation by a trained assessor. A marginal difference (around 10%) between mother-reported outcomes and qualified assessor’s measured outcomes raises a question over the justification of the added effort of on-site observation by a qualified external assessor. In about 5% cases, on-site observation by an assessor was not able to verify the mother-reported achievement; whereas in 3% and 6% of intervention and control children, respectively, on-site observation measured an achievement missed by the mother.

### Implications for practice

In most reported experiences in low and middle-income countries, community-based ECD care was delivered either at households or at day-care centres.^33–39^ This trial has been an effort to develop and test an alternate, clinic-based delivery of ECD care in poor urban populations of a low-middle income country. The intervention was designed to keep the care protocols compatible with a routine healthcare setting. However, the care delivery required a relatively active role on the part of mothers to access the health promotion care at the clinics. The process evaluation has shown that clinic-based delivery of ECD care is feasible for providers, and acceptable for mothers and families living in poor urban localities.^30^

In conclusion, contextualised ECD care, when delivered by non-specialist staff at GP clinics in poor urban localities, can effectively reduce the developmental delays during the first 12 months of child life. The effectiveness and feasibility^30^ of ECD care at GP clinics indicates a possibility of scaling the intervention in other parts of the country.

## Trial registration

The trial is registered with the ISRCTN Current Controlled Trials (ISRCTN48032200).
